# Gait Velocity Alterations in Essential Tremor: a Meta-Analysis

**DOI:** 10.1007/s12311-024-01763-1

**Published:** 2024-12-13

**Authors:** Kenneth Harrison, Brandon M. Peoples, Keven G. Santamaria Guzman, Emily J. Hunter, Harrison C. Walker, Jaimie A. Roper

**Affiliations:** 1 Locomotor and Movement Control Lab, School of Kinesiology, Auburn University, Auburn, AL USA; 2 Department of Neurology, Heersink School of Medicine, University of Alabama at Birmingham, Birmingham, AL USA

**Keywords:** Cerebellum, Locomotion, Movement disorders, Tremor, Ataxia, Fall risk

## Abstract

Essential tremor (ET) is a prevalent movement disorder that impairs gait function, including gait speed - a critical marker of mobility disability and adverse outcomes. This meta-analysis aimed to quantify differences in gait speed between individuals diagnosed with ET compared to people without a movement disorder diagnosis. Electronic databases were searched for studies comparing gait speed in ET patients and controls. Effect sizes were calculated using standardized mean differences (Hedges’ g) and pooled using a random-effects model. Eight studies (390 ET, 227 controls) were included. ET patients exhibited significantly slower gait speeds than controls. The effect size (Hedges’ g = -1.06, 95% CI -1.47 to -0.65, *p* < .001) indicates a large, clinically significant difference. Substantial study heterogeneity was observed (I^2^ = 76.9%). These findings suggest that gait speed deficits are a significant feature of ET, potentially reflecting cerebellar dysfunction. This highlights the need for gait assessment and targeted interventions in ET management to reduce fall risk and improve quality of life. Understanding the moderating factors such as medication type and state, disorder severity, and age could provide significant benefits in the treatment and management of ET.

**Supplementary Information:**

The online version contains supplementary material available at 10.1007/s12311-024-01763-1.

## Introduction

Essential tremor (ET) is a progressive neurological disorder characterized by involuntary rhythmic tremors predominantly affecting the upper extremities, although it can also involve other body regions, including the head, trunk, and lower limbs [1–3]. With an estimated prevalence of around 1% in the general population, ET is one of the most common movement disorders worldwide [4]. However, its exact pathophysiology remains unclear with accumulating evidence suggesting the involvement of the cerebellum and its connections with other brain regions, leading to disruptions in neural circuits responsible for motor control and coordination [5–7]. Therefore, studies that examine the motor comorbidities of ET are vital to better understand its pathophysiology.

The clinical manifestation of ET extends beyond the characteristic tremors, with emerging research highlighting impairments in gait and mobility [8–12] leading to an increased incidence of falls and fall related injuries [13, 14]. Gait is essential for independent ambulation and quality of life, but it is also a complex motor task that requires coordination between multiple neural systems and musculoskeletal components [15, 16]. Existing literature has reported various gait abnormalities in individuals with ET, including reduced gait speed, shorter stride length, and increased stride time variability [8, 11, 17]. Gait speed in particular is critical, as it has been termed the “sixth vital sign” due to its strong associations with functional status, quality of life, and survival in older adults [18, 19].

Reduced gait speed is linked consistently with increased risk of adverse outcomes, including falls, disability, hospitalization, and mortality [19–21], and it is also associated with cognitive impairment, frailty, and chronic conditions such as cardiovascular disease and stroke [19, 22, 23]. Therefore, gait speed is being increasingly seen as a sensitive marker of overall health and well-being, integrating various physiological systems, including musculoskeletal, neurological, and cardiovascular functions [23, 24].

While individual studies report gait impairments in ET, results have been inconsistent with respect to symptom severity and the specific parameters that are affected. This meta-analysis seeks to quantitatively synthesize existing data and (1) to provide a more precise estimate of gait speed deficits in ET and their clinical significance, (2) to identify knowledge gaps, and (3) to guide future research directions. The primary aim of this meta-analysis is to evaluate the differences in self-selected gait speed between individuals with ET and age-matched controls. Given the heterogeneity in the existing literature and the importance of gait function, including gait speed, in maintaining independence and quality of life, a comprehensive meta-analysis is warranted to synthesize the available evidence and provide a quantitative assessment of gait characteristics in individuals with ET compared to healthy controls.

## Methods

### Search Strategy and Data Extraction

**Fig. 1:**
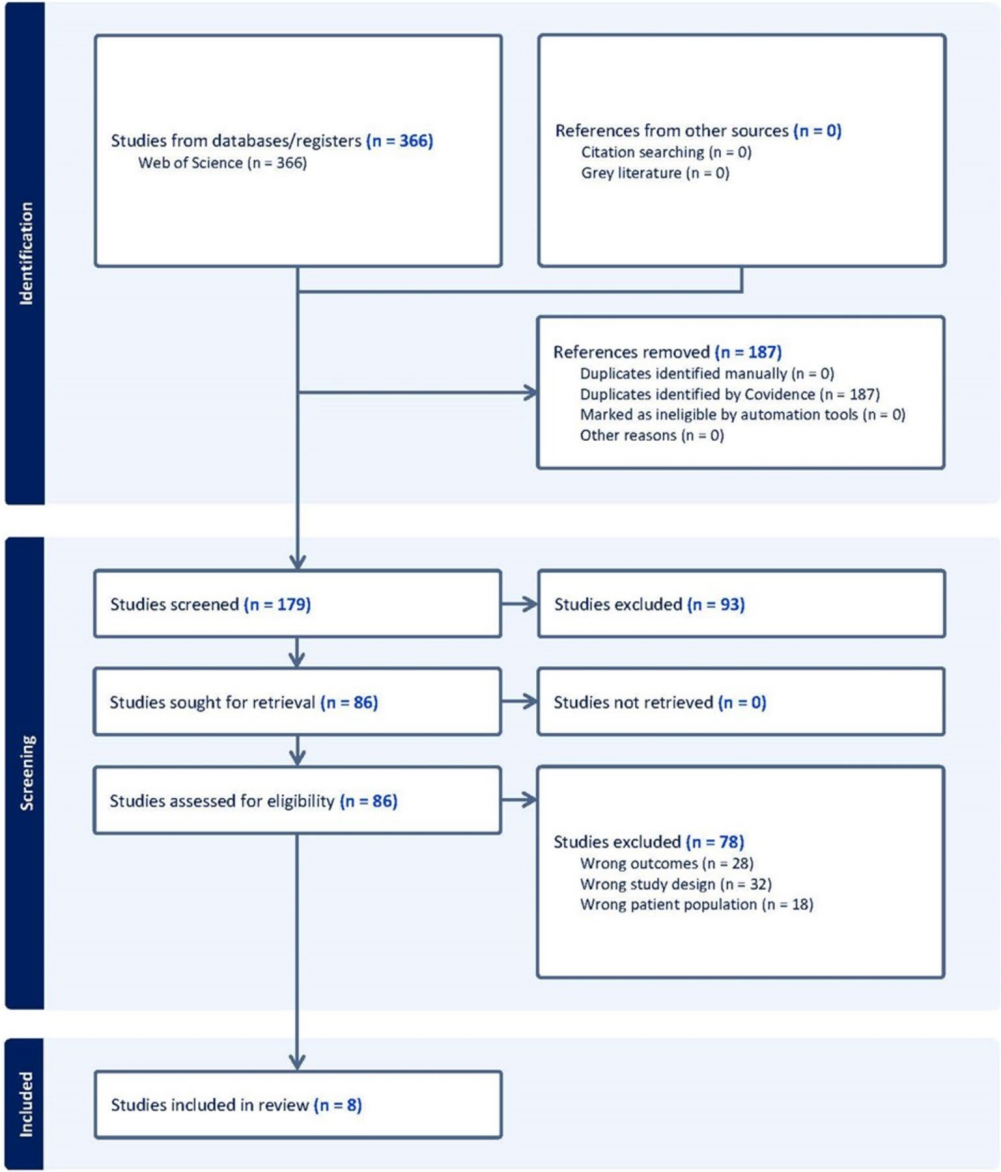
PRISMA diagram

This meta-analysis was conducted according to the 2020 Preferred Reporting Items for Systematic Reviews and Meta-Analysis (PRISMA) guidelines [25]. A comprehensive literature search was conducted in several electronic databases through Web of Science (Clavariate) to identify studies that reported gait speed measurements in separate groups (ET vs control). The search terms included combinations of keywords related to gait speed (e.g., “gait speed,” “walking speed,” “gait velocity”) and group identifiers (e.g., “Essential Tremor”, “control,” “patient,” “disorder”). Studies were included if they (a) reported gait speed measurements in individuals that have been diagnosed with ET as well as controls and (b) provided sufficient data to calculate effect size (e.g., means and standard deviations for each group). Studies were excluded if they were formatted as review articles, case reports, or conference abstracts (Fig. 1). The PRISMA diagram used in Fig. 1 was generated using Covidence’s flow diagram tool. Two authors (KH, EH) independently screened the titles and abstracts of the retrieved records and assessed the full texts of potentially eligible studies for inclusion. Disagreements were resolved through discussion and consensus. For each included study, the following data were extracted: author names, publication year, study design, sample characteristics (group labels, sample sizes, age, sex), gait speed, and relevant statistical information (means, standard deviations, or other data required for effect size calculation).

### Meta-Analytic Procedures

The primary effect size measure was the standardized mean difference (Hedges’ g) in gait speed between groups. For studies reporting means and standard deviations, Hedges’ g was calculated as the difference between the group means divided by the pooled standard deviation, with a correction factor applied to account for potential bias due to small sample sizes [26]. Negative effect sizes indicate slower gait speed in the patient group compared to the healthy control group. A random-effects meta-analysis model was used to combine the effect sizes from individual studies, accounting for potential heterogeneity in actual effects across studies [27]. Heterogeneity was assessed using the Q statistic and I² index. Data extracted from each study was stored in Microsoft Excel for Microsoft 365 MSO (Version 2403 Build 16.0.17425.20176) 64-bit. All statistical analyses and visualizations in this meta-analysis were conducted in R studio (Posit Software, version 2023.12.0, PBC, Build 369) using R (version 4.3.1) [28] and the metafor package (version 4.6.0) [29].

## Results

### Search Results

A Total of 452 studies were found in the initial search. After removal of duplicates, 180 studies remained (Fig. 1). Using the Covidence Systematic Review tool, two reviewers rated studies based on exclusion criteria and only studies that were agreed upon by both reviewers were included. After the abstract and title screening, we were left with seventy-five studies. Only 8 of these studies ended up being fit for data extraction [9, 10, 30–35].

### Study Characteristics

The meta-analysis included eight studies with a sample size of 617 (227 control and 390 with ET) conducted between the years 2001–2022 across the Unites States, Czech Republic, and Germany (Table 1). Sample sizes ranged from 24 ET patients in Fernandez et al. [31] to 151 in Rao et al. [32]. The included studies consistently reported reduced gait speed in ET patients compared to controls, with varying degrees of impairment. Study designs ranged from simple clinical assessments to sophisticated 3D motion capture analyses. The mean age of ET participants varied across studies, from 50.3 ± 21.1 years in Stolze et al. [35] to 86.0 ± 4.6 years in Rao et al. [10]. Five studies used overground walking protocols [9, 10, 31, 32, 34], two used treadmill walking [30, 35], and one incorporated both [31]. Six studies assessed both normal and tandem gait, while two focused solely on normal gait. Gait assessment methods varied, including clinical rating scales, pressure-sensitive walkways, and 3D motion capture systems. Common outcome measures across studies were gait speed, step width, and measures of gait variability. All studies reported reduced gait speed in ET patients compared to controls, with effect sizes ranging from − 0.5 to −1.8. Six studies found increased step width in ET patients, and five reported increased gait variability. Three studies included additional comparison groups: two compared ET to Parkinson’s disease [31, 34], and one examined the effects of deep brain stimulation in ET patients [30].

**Table 1:** Study details

Authors	Year	Treatment	Control
Skinner et al.	2022	24	20
Rao et al.	2013	161	62
Fernandez et al.	2013	24	38
Rao et al.	2011	104	40
Hoskovcová et al.	2013	30	25
Roemmich et al.	2013	11	11
Stolze et al.	2001	25	21
Fasano et al.	2010	11	10

### Meta Analysis

**Fig. 2:**
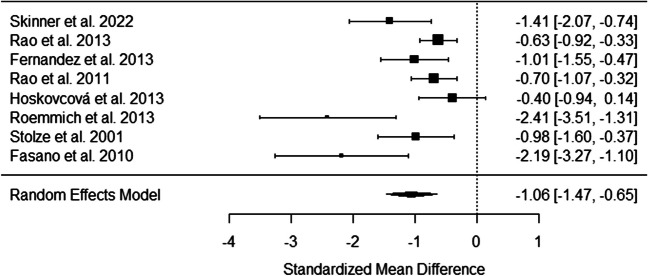
Forest Plot

**Fig. 3:**
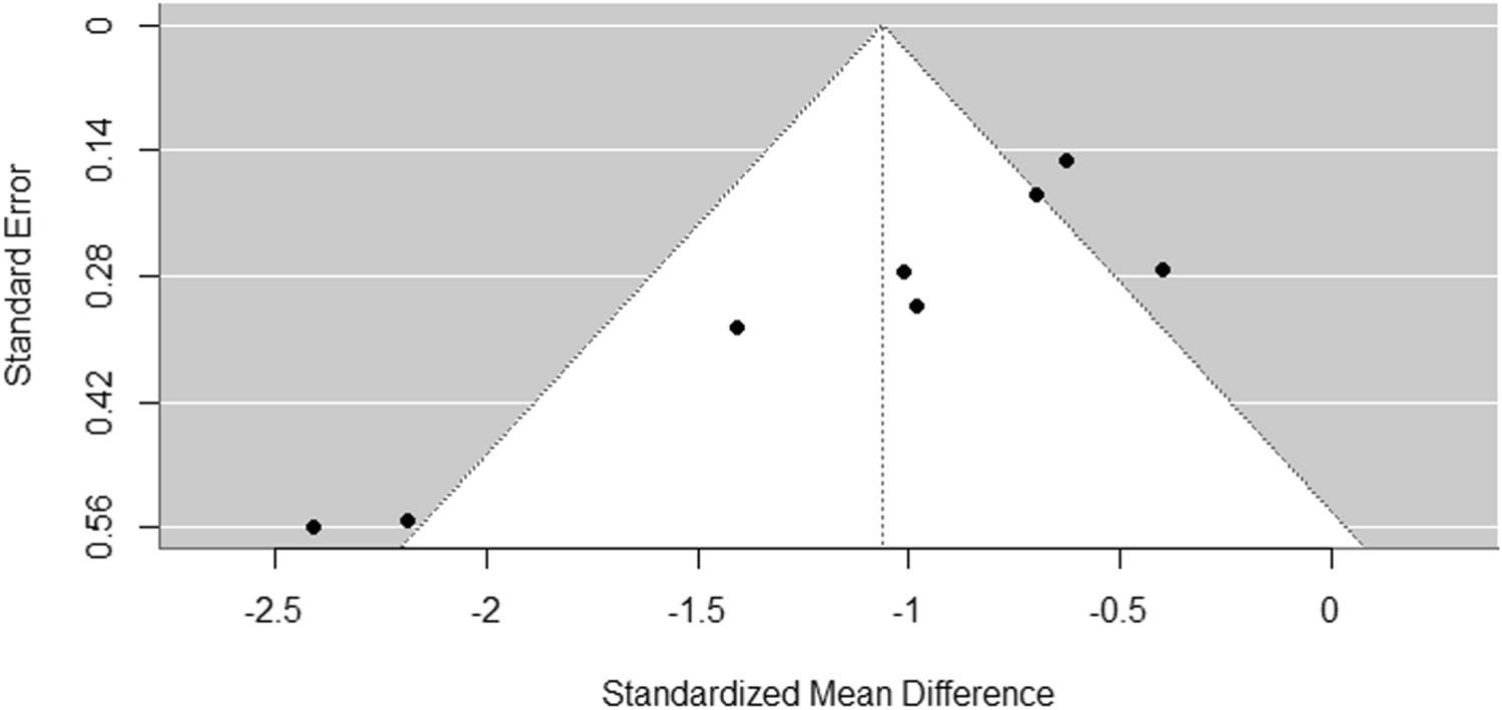
Funnel Plot

The forest plot in Fig. 2 displays the individual study effect sizes and 95% confidence intervals for the difference in gait speed between those with ET and controls. The random-effects meta-analysis revealed a significant overall effect. Individuals diagnosed with ET exhibited slower gait speeds compared to controls (pooled Hedges’ g = −1.06, 95% CI (−1.47, −0.65), *p* < .001) which is shown in the forest plot (Fig. 2). There was evidence of substantial heterogeneity across the included studies, as indicated by the Q statistic (Q (7) = 22.34, *p* < .01) and the I² index (76.92%), suggesting that almost 80% of the observed variance in effect sizes was due to true heterogeneity rather than random chance. While a moderator or meta regression analysis would have been useful to further identify study groupings, we did not have enough studies to properly perform or interpret these tests [36]. To assess potential publication bias, a funnel plot was created (Fig. 3). The funnel plot displays the relationship between study effect sizes and their standard errors. In the absence of publication bias, the plot should resemble a symmetrical inverted funnel. Visual inspection of our funnel plot suggests a potential inverted funnel shape forming with a peak being reached at around − 0.5 mean difference, and error increasing as we move away from this mean difference value. The two studies with lowest error also had the highest sample size, and were both done in the same lab [10, 32].

## Discussion

This meta-analysis provides the first quantitative synthesis of gait speed differences between individuals with essential tremor (ET) and healthy controls. Our primary findings were: (1) Individuals with ET exhibit significantly slower gait speeds compared to controls, with a large effect size (Hedges’ g = −1.06, 95% CI −1.47 to −0.65, *p* < .001); (2) There was substantial heterogeneity across studies (I² = 76.92%), indicating variability in the magnitude of gait speed alterations among ET populations. Across the eight studies included in the analysis, our findings align with and extend previous research demonstrating gait abnormalities in ET. The large effect size we observed suggests that gait speed reduction is a clinically significant feature of ET, consistent with studies reporting various gait impairments in this population [8, 11, 37]. The magnitude of gait speed deficit observed is comparable to or greater than that seen in other neurological disorders, underscoring the importance of gait dysfunction in ET [38]. Gait speed relies on the integrated function of multiple physiological systems, including musculoskeletal, neuromuscular, cardiopulmonary, and cognitive domains [23]. The observed deficits in gait speed among those with ET could have several mechanisms responsible such as neuromuscular impairments, cortico-thalamic dysfunction, or cerebellar dysfunction leading to increased cognitive/attentional demands for locomotion [8, 11, 39].

The involuntary rhythmic tremors that characterize ET can directly impair the neuromuscular control required for smooth, coordinated gait patterns. Tremors affecting the lower extremities can disrupt the precise timing and force generation needed for proper push-off forces and stance stability during the gait cycle [3, 10]. Similarly, axial tremors of the trunk can compromise dynamic balance and increase postural sway while walking [9]. Individuals with ET may adopt abnormal gait patterns and biomechanical adjustments to compensate for tremors and maintain stability during gait. Common compensatory mechanisms include shortening stride length, widening the base of support, and adopting a stooped, cautious posture [11, 17, 40]. While aimed at prioritizing stability over mobility, these altered gait kinematics can inadvertently reduce overall gait speed [41].

Our research on ET pathophysiology suggests that cerebellar dysfunction may play a role in explaining these gait impairments, although the exact mechanisms remain to be fully elucidated. The cerebellum, with its crucial role in motor coordination, balance, and movement timing, is a key component of gait [42–44]. Neuroimaging studies have shown structural and functional abnormalities in the cerebellum of ET patients [6]. While cerebellar dysfunction could contribute to the gait speed reductions observed in our meta-analysis, it is important to note that our data do not directly address this mechanistic hypothesis. These findings further underscore the importance of our research in understanding the mechanisms of gait impairments in ET. An alternative perspective suggests that ET may primarily involve cortico-thalamic dysfunction, with bottom-up modulation from the cerebellar fibers projecting into the ventral intermediate nucleus. This view aligns with the observation that tremor oscillations might be too rapid to be solely mediated by cerebellar loops, whereas the slower process of ataxia could be more directly influenced by cerebellar function [35]. Furthermore, ET is increasingly viewed as a network disorder involving disrupted oscillatory activity in the cerebello-thalamo-cortical circuit [7, 45]. These pathways are critical for the precise timing and execution of motor commands. Abnormal activity in this network could affect tremor generation and the complex neural control required for efficient gait. Emerging research highlights gait’s cognitive and attentional demands as well, particularly in neurological deficits like tremor [15, 39]. The processes of motor planning, updating sensory feedback, allocating attentional resources, and implementing online corrections during gait rely on integrated neural networks involving cortical and subcortical regions [46, 47]. As suggested by our findings, the increased cognitive demand of walking for those with ET may reflect the need for greater cortical involvement to compensate for dysfunctional automatic control from subcortical circuits. These central nervous system mechanisms may interact with peripheral factors, such as tremors in the lower limbs and trunk, to produce the observed gait impairments. Further research is needed to clarify the specific contributions of cerebellar, thalamic, and cortical dysfunction to gait impairments in ET.

The mechanisms we’ve identified may interact synergistically to compound mobility limitations and elevate fall risk in those with ET. Reduced gait speed itself is an established risk factor for falls, which can precipitate further functional decline [23, 48]. The gait slowing observed in ET may significantly impair an individual’s ability to respond quickly and maintain balance in destabilizing situations. Furthermore, abnormal gait patterns may restrict mechanisms for dynamic balance recovery after perturbations [49, 50]. The convergence of these gait impairments elevates the propensity for falls and related injurious events in ET, which can catalyze a vicious cycle of increasing frailty, sedentary behavior, and accelerated functional deterioration [13, 14, 51, 52].

Our findings highlight the urgent need for routine gait speed monitoring and targeted interventions to improve mobility function as prudent fall prevention strategies in this clinical population at heightened fall risk. Clinicians should consider incorporating gait speed tests into routine ET examinations, as this could provide valuable prognostic information and guide treatment decisions. Traditional rehabilitation approaches for neurological gait disorders may need to be adapted to address the specific challenges faced by ET patients. For instance, dual-task training that combines gait exercises with cognitive tasks could help improve the allocation of attentional resources during walking, potentially mitigating the cognitive-motor interference observed in ET [32]. Fall prevention programs should be tailored to address the specific gait impairments identified in ET, such as reduced speed and increased variability. Additionally, interventions focusing on improving lower limb strength, balance, and coordination may be beneficial. Incorporating technology like wearable sensors for gait monitoring and biofeedback could provide valuable tools for both assessment and intervention in clinical and home settings.

While our findings demonstrate significant gait speed deficits in ET, it is important to note that the relationship between lower limb tremor and gait difficulties remains poorly understood. Lower limb tremor is not commonly reported or systematically assessed in clinical settings, which may lead to an underestimation of its prevalence and impact on gait. This gap in our understanding has led to differing perspectives within the scientific community. One view aligns with the recent ‘ET-plus’ classification, which suggests that ET can involve a broader spectrum of motor and non-motor symptoms beyond the classic upper limb tremor [1]. Proponents of this view argue that gait difficulties, as demonstrated in our meta-analysis, would fall under this ‘ET-plus’ category, suggesting that they may be part of a more systemic manifestation of the disorder rather than solely a consequence of lower limb tremor. However, there is significant debate surrounding the ‘ET-plus’ concept. Critics argue that this classification may lead to an overly broad and potentially misleading characterization of ET and contend that many of the additional symptoms attributed to ‘ET-plus’ may represent comorbidities or age-related changes rather than intrinsic features of ET itself. These researchers emphasize the importance of maintaining a more focused definition of ET to avoid diagnostic confusion and ensure targeted research and treatment approaches [53, 54].

Given these conflicting perspectives, it is crucial for future studies to systematically assess lower limb tremor alongside gait parameters to elucidate potential relationships and mechanisms. Such research could provide valuable insights into whether gait impairments in ET are primarily driven by lower limb tremor, cerebellar dysfunction, or other factors. Additionally, longitudinal studies tracking the progression of symptoms in ET patients could help clarify whether additional features emerge as part of the disease process or as separate, albeit potentially related, conditions. This ongoing debate underscores the complexity of ET and the need for continued rigorous research. A nuanced understanding of the relationship between ET and associated symptoms is crucial for developing targeted interventions to improve gait, reduce fall risk, and enhance overall quality of life for individuals with ET. As the field progresses, it will be important to critically evaluate new evidence and remain open to refining our conceptualization of ET based on robust scientific findings. Future studies should aim to systematically assess lower limb tremor alongside gait parameters to elucidate potential relationships and mechanisms. Such research could provide valuable insights into whether gait impairments in ET are primarily driven by lower limb tremor, cerebellar dysfunction, or other factors associated with the broader ‘ET-plus’ phenotype. This understanding is crucial for developing targeted interventions to improve gait and reduce fall risk in individuals with ET.

While pharmacological interventions like propranolol and primidone are primarily aimed at reducing tremor amplitude, they may not adequately address gait impairments. The significant gait speed deficits revealed in our meta-analysis highlight the importance of considering potential impacts on gait when planning treatments for ET. While gait may not be a primary therapeutic target due to limited gait-specific therapies in ET, clinicians should be mindful of how various treatments might affect gait and balance. Deep brain stimulation (DBS) of the ventral intermediate nucleus of the thalamus has shown promise in improving tremor symptoms, but its effects on gait in ET are less clear and sometimes contradictory. Fasano et al. [30] reported improvements in some gait parameters post-DBS, particularly in patients with pre-existing gait ataxia. However, Roemmich et al. [44] found that gait actually worsened immediately following DBS surgery, potentially due to a microlesion effect. This discrepancy highlights the complex relationship between tremor control and gait function in ET. Recent research has explored alternative DBS targets that might better address both tremor and gait symptoms. For instance, Barbe et al. [55] investigated the effects of posterior subthalamic area stimulation on gait in ET patients, finding potential improvements in both tremor and certain gait parameters. However, the long-term effects of such interventions on gait speed specifically remain to be fully elucidated.

The magnitude of gait speed deficits observed in our meta-analysis (pooled Hedges’ g = −1.06) underscores the need for treatment approaches that specifically target gait impairments in ET. This might involve combination therapies that address both tremor and gait symptoms. For example, coupling traditional pharmacological or surgical interventions with targeted gait rehabilitation could potentially yield better functional outcomes. Emerging non-invasive brain stimulation techniques, such as transcranial magnetic stimulation (TMS) or transcranial direct current stimulation (tDCS), warrant further investigation as potential adjunct therapies. These methods could potentially modulate cerebellar activity and improve gait function without the risks associated with surgical interventions [56]. Our results also highlight the importance of comprehensive outcome assessments in ET treatment studies.

## Limitations

While the meta-analytic findings were robust in showing an overall effect of ET on gait slowing, there was substantial and statistically significant heterogeneity across studies (Q = 22.34, *p* < .01). The high degree of heterogeneity (I^2^ = 76.92%) indicates that a sizeable proportion of the observed variance in effect sizes was due to actual between-study differences rather than sampling variability alone. This suggests the presence of unmeasured study-level moderators contributing to variability in the effect of ET on gait speed across studies. The high degree of heterogeneity observed suggests that factors such as disorder severity, age, medication status, and specific gait assessment protocols may significantly influence results. This heterogeneity limits our ability to draw definitive conclusions about the exact magnitude of gait speed deficits across all ET patients. For instance, patients with more severe axial or lower limb tremor might exhibit greater gait speed deficits. Similarly, those with longer disorder duration or older age of onset might show more pronounced gait impairments due to cumulative neurological changes or age-related factors. Future research should aim to identify these potential subgroups and their associated characteristics, as this could lead to more personalized treatment and rehabilitation approaches. Given the small number of included studies (k = 8) and the lack of statistical power, we could not systematically evaluate these potential moderating influences through subgroup analysis or meta-regression techniques [36]. The funnel plot (Fig. 3) provides insight into potential publication bias in our meta-analysis with the relative symmetry of the plot suggesting publication bias is unlikely to be a major concern. It is important to note that visual interpretation of funnel plots can be subjective, especially with a small number of studies. Therefore, these results should be interpreted cautiously.

Additional limitations of the present work include the small number of studies eligible for inclusion and the low sample variability, with two studies representing most of the sample (Table 1). Further, our analysis focused exclusively on the gait speed metric, which, while well-established, may only partially capture the multidimensional nature of gait impairments linked to ET. All studies included in this analysis were also cross-sectional in nature which limits our ability to draw conclusions on longitudinal changes in individuals with ET. An important limitation of this meta-analysis, and indeed of the current literature on gait impairments in Essential Tremor (ET), is the lack of patient-reported outcomes regarding gait and balance. Upon review, one of the eight studies included in our analysis explicitly reported on subjective patient experiences or comments related to gait and balance difficulties [9]. This gap highlights a potential disconnect between objectively measured gait parameters and patients’ lived experiences of gait and balance impairments. As such, our meta-analysis may not fully capture the spectrum of gait-related challenges faced by individuals with ET in their daily lives. There may be subtle or context-specific gait and balance issues that are significant to patients but are not adequately detected or quantified by standardized gait assessments. This limitation underscores the need for future studies to incorporate both objective measures and patient-reported outcomes to provide a more comprehensive understanding of gait impairments in ET.

### Future Directions

Looking ahead, larger-scale meta-analyses with more primary studies permit a more granular investigation of essential moderators that could explain sources of heterogeneity. Such work would enhance our understanding of which patient subgroups or characteristics are linked to greater versus lesser gait speed impairments in ET. Future studies should aim to standardize gait assessment protocols in ET with comparable methodology (age, duration, and severity of tremor etc.) to facilitate more direct comparisons and determine how findings might change across age groups. Longitudinal studies are needed to track the evolution of gait kinematics in ET with additional measurement of subjective gait and balance experiences. Additionally, research combining gait analysis with neuroimaging could provide valuable insights into the neural correlates of gait impairments in ET, particularly focusing on cerebellar circuits. This knowledge could guide more personalized prognostic estimates and targeted interventions. The evaluation of therapeutic approaches designed to improve gait speed represents another critical area for future randomized trials and meta-analyses in this population.

## Conclusion

In summary, this meta-analysis demonstrates ET’s clear and negative impact on gait speed compared to healthy controls. The pooled effect size highlights clinically meaningful mobility impairments, likely contributing to downstream disability, falls, and loss of independence often observed in this group [13]. While future larger-scale meta-analytic work is needed to delineate key moderating factors, identify high-risk subgroups, and evaluate therapeutic interventions, the current findings reinforce gait speed as an essential clinical marker and potential treatment target for preserving mobility and function in patients diagnosed with ET.

## Supplementary Information

Below is the link to the electronic supplementary material.

ESM 1 (XLSX 9.98 KB)

## Data Availability

Data is provided within the manuscript or supplementary information files.

## References

[1] Bhatia KP, Bain P, Bajaj N, Elble RJ, Hallett M, Louis ED, Raethjen J, Stamelou M, Testa CM, Deuschl G, Tremor Task Force of the International Parkinson and Movement Disorder Society. Consensus statement on the classification of tremors. From the task force on tremor of the International Parkinson and Movement Disorder Society. Mov Disorders: Off J Mov Disorder Soc. 2018;33(1):75–87. 10.1002/mds.27121. 10.1002/mds.27121 PMC6530552 29193359

[2] Hopfner F, Ahlf A, Lorenz D, Klebe S, Zeuner KE, Kuhlenbäumer G, Deuschl G. Early- and late-onset essential tremor patients represent clinically distinct subgroups. Mov Disorders: Official J Mov Disorder Soc. 2016;31(10):1560–6. 10.1002/mds.26708. 10.1002/mds.26708 27384030

[3] Roper JA, Brinkerhoff SA, Harrison BR, Schmitt AC, Roemmich RT, Hass CJ. Persons with essential tremor can adapt to new walking patterns. J Neurophysiol. 2019a;122(4):1598–605. 10.1152/jn.00320.2019. 31365318 10.1152/jn.00320.2019

[4] Louis ED, Ferreira JJ. How common is the most common adult movement disorder? Update on the worldwide prevalence of essential tremor. Mov Disorders: Official J Mov Disorder Soc. 2010;25(5):534–41. 10.1002/mds.22838. 10.1002/mds.22838 20175185

[5] Benito-León J, Louis E. Update on essential tremor. Minerva Med. 2011;102(6):417–40. 22193375

[6] Cerasa A, Messina D, Nicoletti G, Novellino F, Lanza P, Condino F, Arabia G, Salsone M, Quattrone A. Cerebellar atrophy in essential tremor using an automated segmentation method. AJNR Am J Neuroradiol. 2009;30:1240–3. 10.3174/ajnr.A1544. 19342539 10.3174/ajnr.A1544 PMC7051361

[7] Raethjen J, Deuschl G. The oscillating central network of essential tremor. Clin Neurophysiol. 2012;123(1):61–4. 10.1016/j.clinph.2011.09.024. 22055842 10.1016/j.clinph.2011.09.024

[8] Cabaraux P, Agrawal S, Cai H, Calabro R, Carlo C, Loic D, Sarah D, Habas C, Horn A, Ilg W, Louis E, Mitoma H, Monaco V, Petracca M, Ranavolo A, Rao A, Ruggieri S, Schirinzi T, Serrao M, Manto M. Consensus Paper: Ataxic Gait. Cerebellum. 2023;22(3):394–430. 10.1007/s12311-022-01373-9. 35414041 10.1007/s12311-022-01373-9

[9] Hoskovcová M, Ulmanová O, Šprdlík O, Sieger T, Nováková J, Jech R, Růžička E. Disorders of balance and gait in essential tremor are associated with midline tremor and age. Cerebellum. 2013;12(1):27–34. 10.1007/s12311-012-0384-4. 22535593 10.1007/s12311-012-0384-4

[10] Rao AK, Gillman A, Louis ED. Quantitative gait analysis in essential tremor reveals impairments that are maintained into advanced age. Gait Posture. 2011;34(1):65–70. 10.1016/j.gaitpost.2011.03.013. 21478017 10.1016/j.gaitpost.2011.03.013 PMC3575132

[11] Roper JA, Terza MJ, De Jesus S, Jacobson CE, Hess CW, Hass CJ. Spatiotemporal gait parameters and tremor distribution in essential tremor. Gait Posture. 2019b;71:32–7. 10.1016/j.gaitpost.2019.04.004. 31004995 10.1016/j.gaitpost.2019.04.004

[12] Zubair A, Cersonsky TEK, Kellner S, Huey ED, Cosentino S, Louis ED. What predicts mortality in essential tremor? A prospective, longitudinal study of elders. Front Neurol. 2018;9. 10.3389/fneur.2018.01077. 10.3389/fneur.2018.01077 PMC6292933 30581416

[13] Gerbasi ME, Nambiar S, Reed S, Hennegan K, Hadker N, Eldar-Lissai A, Cosentino S. Essential tremor patients experience significant burden beyond tremor: A systematic literature review. Front Neurol. 2022;13. 10.3389/fneur.2022.891446 10.3389/fneur.2022.891446 PMC9354397 35937052

[14] Kobayashi K, Imagama S, Inagaki Y, Suzuki Y, Ando K, Nishida Y, Nagao Y, Ishiguro N. Incidence and characteristics of accidental falls in hospitalizations. Nagoya J Med Sci. 2017;79(3):291–8. 10.18999/nagjms.79.3.291. 28878434 10.18999/nagjms.79.3.291 PMC5577015

[15] Mirelman A, Bonato P, Camicioli R, Ellis TD, Giladi N, Hamilton JL, Hass CJ, Hausdorff JM, Pelosin E, Almeida QJ. Gait impairments in Parkinson’s disease. Lancet Neurol. 2019;18(7):697–708. 10.1016/S1474-4422(19)30044-4. 30975519 10.1016/S1474-4422(19)30044-4

[16] Nutt JG, Bloem BR, Giladi N, Hallett M, Horak FB, Nieuwboer A. Freezing of gait: moving forward on a mysterious clinical phenomenon. Lancet Neurol. 2011;10(8):734–44. 10.1016/S1474-4422(11)70143-0. 21777828 10.1016/S1474-4422(11)70143-0 PMC7293393

[17] Hoskovcová M, Dušek P, Sieger T, Brožová H, Zárubová K, Bezdíček O, Šprdlík O, Jech R, Štochl J, Roth J, Růžička E. Predicting falls in Parkinson Disease: what is the value of instrumented testing in OFF medication state? PLoS ONE. 2015;10(10):e0139849. 10.1371/journal.pone.0139849. 26443998 10.1371/journal.pone.0139849 PMC4596567

[18] Fritz S, Lusardi M. White paper: walking speed: the sixth vital sign. J Geriatr Phys Ther (2001). 2009;32(2):46–9. 20039582

[19] Studenski S, Perera S, Patel K, Rosano C, Faulkner K, Inzitari M, Brach J, Chandler J, Cawthon P, Connor EB, Nevitt M, Visser M, Kritchevsky S, Badinelli S, Harris T, Newman AB, Cauley J, Ferrucci L, Guralnik J. Gait speed and survival in older adults. JAMA. 2011;305(1):50–8. 10.1001/jama.2010.1923. 21205966 10.1001/jama.2010.1923 PMC3080184

[20] van Abellan G, Rolland Y, Andrieu S, Bauer J, Beauchet O, Bonnefoy M, Cesari M, Donini LM, Guyonnet G, Inzitari S, Nourhashemi M, Onder F, Ritz G, Salva P, Visser A, M., Vellas B. Gait speed at usual pace as a predictor of adverse outcomes in community-dwelling older people an International Academy on Nutrition and Aging (IANA) Task Force. J Nutr Health Aging. 2009;13(10):881–9. 10.1007/s12603-009-0246-z.19924348 10.1007/s12603-009-0246-z

[21] Perera T, Yohanandan SAC, Thevathasan W, Jones M, Peppard R, Evans AH, Tan JL, McKay CM, McDermott HJ. Clinical validation of a precision electromagnetic tremor measurement system in participants receiving deep brain stimulation for essential tremor. Physiol Meas. 2016;37(9):1516–27. 10.1088/0967-3334/37/9/1516. 27511464 10.1088/0967-3334/37/9/1516

[22] Inzitari M, Newman AB, Yaffe K, Boudreau R, de Rekeneire N, Shorr R, Harris TB, Rosano C. Gait speed predicts decline in attention and psychomotor speed in older adults: the health aging and body composition study. Neuroepidemiology. 2007;29(3–4):156–62. 10.1159/000111577. 18042999 10.1159/000111577 PMC2824580

[23] Peel NM, Alapatt LJ, Jones LV, Hubbard RE. The association between gait speed and cognitive status in community-dwelling older people: a systematic review and meta-analysis. J Gerontol A. 2019;74(6):943–8. 10.1093/gerona/gly140. 10.1093/gerona/gly140 29917045

[24] Middleton A, Fritz SL, Lusardi M. Walking speed: the functional vital sign. J Aging Phys Act. 2015;23(2):314–22. 10.1123/japa.2013-0236. 24812254 10.1123/japa.2013-0236 PMC4254896

[25] Page MJ, McKenzie JE, Bossuyt PM, Boutron I, Hoffmann TC, Mulrow CD, Shamseer L, Tetzlaff JM, Akl EA, Brennan SE, Chou R, Glanville J, Grimshaw JM, Hróbjartsson A, Lalu MM, Li T, Loder EW, Mayo-Wilson E, McDonald S, Moher D. The PRISMA 2020 statement: an updated guideline for reporting systematic reviews. BMJ. 2021;372:n71. 10.1136/bmj.n71. 33782057 10.1136/bmj.n71 PMC8005924

[26] Hedges L, Olkin I. (1985). Statistical methods in meta-analysis. In Stat Med (Vol. 20). 10.2307/1164953.

[27] Borenstein M, Hedges LV, Higgins JPT, Rothstein HR. A basic introduction to fixed-effect and random-effects models for meta-analysis. Res Synth Methods. 2010;1(2):97–111. 10.1002/jrsm.12. 10.1002/jrsm.12 26061376

[28] R Core Team. R: a language and environment for statistical computing. Vienna: R Foundation for Statistical Computing; 2021. https://www.R-project.org/.

[29] Viechtbauer W. Conducting meta-analyses in R with the metafor package. J Stat Softw. 2010;36:1–48. 10.18637/jss.v036.i03.

[30] Fasano A, Herzog J, Raethjen J, Rose FEM, Muthuraman M, Volkmann J, Falk D, Elble R, Deuschl G. Gait ataxia in essential tremor is differentially modulated by thalamic stimulation. Brain. 2010;133(12):3635–48. 10.1093/brain/awq267. 20926368 10.1093/brain/awq267

[31] Fernandez KM, Roemmich RT, Stegemöller EL, Amano S, Thompson A, Okun MS, Hass CJ. Gait initiation impairments in both essential tremor and Parkinson’s disease. Gait Posture. 2013;38(4):956–61. 10.1016/j.gaitpost.2013.05.001. 23726428 10.1016/j.gaitpost.2013.05.001 PMC3778167

[32] Rao AK, Uddin J, Gillman A, Louis ED. Cognitive motor interference during dual-task gait in essential tremor. Gait Posture. 2013;38(3):403–9. 10.1016/j.gaitpost.2013.01.006. 23369662 10.1016/j.gaitpost.2013.01.006 PMC3679258

[33] Roemmich RT, Zeilman PR, Vaillancourt DE, Okun MS, Hass CJ. Gait variability magnitude but not structure is altered in essential tremor. J Biomech. 2013;46(15):2682–7. 10.1016/j.jbiomech.2013.07.039. 24011360 10.1016/j.jbiomech.2013.07.039 PMC3832140

[34] Skinner JW, Lee HK, Hass CJ. Evaluation of gait termination strategy in individuals with essential tremor and Parkinson’s disease. Gait Posture. 2022;92:338–42. 10.1016/j.gaitpost.2021.12.007. 34920358 10.1016/j.gaitpost.2021.12.007

[35] Stolze H, Petersen G, Raethjen J, Wenzelburger R, Deuschl G. The gait disorder of advanced essential tremor. Brain. 2001;124(11):2278–86. 10.1093/brain/124.11.2278. 11673328 10.1093/brain/124.11.2278

[36] Higgins JPT, Green S, editors. Cochrane handbook for systematic reviews of interventions version 5.0.2 [updated September 2009]. The Cochrane Collaboration; 2008. Available from www.cochrane-handbook.org.

[37] Rao AK, Louis ED. Timing control of gait: a study of essential tremor patients vs. age-matched controls. Cerebellum Ataxias. 2016;3(1):5. 10.1186/s40673-016-0043-5. 26937284 10.1186/s40673-016-0043-5 PMC4774137

[38] Zanardi APJ, da Silva ES, Costa RR, Passos-Monteiro E, Dos Santos IO, Kruel LFM, Peyré-Tartaruga LA. Gait parameters of Parkinson’s disease compared with healthy controls: a systematic review and meta-analysis. Sci Rep. 2021;11(1):752. 10.1038/s41598-020-80768-2. 33436993 10.1038/s41598-020-80768-2 PMC7804291

[39] Monaghan PG, Murrah WM, Walker HC, Neely KA, Roper JA. Evaluating postural transition movement performance in individuals with essential tremor via the instrumented timed up and go. Sensors. 2024;24(7):2216. 10.3390/s24072216. 38610427 10.3390/s24072216 PMC11014324

[40] Zesiewicz TA, Elble RJ, Louis ED, Gronseth GS, Ondo WG, Dewey RB, Okun MS, Sullivan KL, Weiner WJ. Evidence-based guideline update: treatment of essential tremor: report of the quality standards subcommittee of the American Academy of Neurology. Neurology. 2011;77(19):1752–5. 10.1212/WNL.0b013e318236f0fd. 22013182 10.1212/WNL.0b013e318236f0fd PMC3208950

[41] Hass CJ, Waddell DE, Wolf SL, Juncos JL, Gregor RJ. Gait initiation in older adults with postural instability. Clin Biomech Elsevier Ltd. 2008;23(6):743–53. 10.1016/j.clinbiomech.2008.02.012. 10.1016/j.clinbiomech.2008.02.012 PMC2954654 18407387

[42] Deuschl G, Elble R. Essential tremor—neurodegenerative or nondegenerative disease towards a working definition of ET. Mov Disorders: Off J Mov Disorder Soc. 2009;24(14):2033–41. 10.1002/mds.22755. 10.1002/mds.22755 19750493

[43] Louis ED, Vonsattel JPG. The emerging neuropathology of essential tremor. Mov Disord. 2008;23(2):174–82. 10.1002/mds.21731. 17999421 10.1002/mds.21731 PMC2692583

[44] Roemmich R, Roper J, Eisinger R, Cagle J, Maine L, Deeb W, Shukla A, Hess C, Gunduz A, Foote K, Okun M, Hass C. Gait worsening and the microlesion effect following deep brain stimulation for essential tremor. J Neurol Neurosurg Psychiatry. 2019;90(8):913–9. 10.1136/jnnp-2018-319723. 30846538 10.1136/jnnp-2018-319723

[45] Hallett M. Tremor: pathophysiology. Parkinsonism Relat Disord. 2014;(20 Suppl 1):S118-122. 10.1016/S1353-8020(13)70029-4 10.1016/S1353-8020(13)70029-4 24262161

[46] Al-Yahya E, Dawes H, Smith L, Dennis A, Howells K, Cockburn J. Cognitive motor interference while walking: a systematic review and meta-analysis. Neurosci Biobehav Rev. 2011;35(3):715–28. 10.1016/j.neubiorev.2010.08.008. 20833198 10.1016/j.neubiorev.2010.08.008

[47] Hamacher D, Herold F, Wiegel P, Hamacher D, Schega L. Brain activity during walking: a systematic review. Neurosci Biobehav Rev. 2015;57:310–27. 10.1016/j.neubiorev.2015.08.002. 26306029 10.1016/j.neubiorev.2015.08.002

[48] Quach L, Galica AM, Jones RN, Procter-Gray E, Manor B, Hannan MT, Lipsitz LA. The nonlinear relationship between gait speed and falls: the maintenance of balance, independent living, intellect, and zest in the elderly of Boston study. J Am Geriatr Soc. 2011;59(6):1069–73. 10.1111/j.1532-5415.2011.03408.x. 21649615 10.1111/j.1532-5415.2011.03408.x PMC3141220

[49] Maki BE. Gait changes in older adults: predictors of falls or indicators of fear. J Am Geriatr Soc. 1997;45(3):313–20. 10.1111/j.1532-5415.1997.tb00946.x. 9063277 10.1111/j.1532-5415.1997.tb00946.x

[50] Menz HB, Lord SR, Fitzpatrick RC. Age-related differences in walking stability. Age Ageing. 2003;32(2):137–42. 10.1093/ageing/32.2.137. 12615555 10.1093/ageing/32.2.137

[51] Delbaere K, Close JCT, Heim J, Sachdev PS, Brodaty H, Slavin MJ, Kochan NA, Lord SR. A multifactorial approach to understanding fall risk in older people. J Am Geriatr Soc. 2010;58(9):1679–85. 10.1111/j.1532-5415.2010.03017.x. 20863327 10.1111/j.1532-5415.2010.03017.x

[52] Terroso M, Rosa N, Torres Marques A, Simoes R. Physical consequences of falls in the elderly: a literature review from 1995 to 2010. Eur Rev Aging Phys Act. 2014;11(1):Article 1. 10.1007/s11556-013-0134-8.

[53] Elble RJ. Estimating change in tremor amplitude using clinical ratings: recommendations for clinical trials. Tremor Other Hyperkinet Mov (N Y). 2018;8:600. 10.7916/D89C8F3C. 10.7916/D89C8F3C PMC6802602 31637097

[54] Louis ED. Essential tremor: a nuanced approach to the clinical features. Pract Neurol. 2019;19(5):389–98. 10.1136/practneurol-2018-002183. 10.1136/practneurol-2018-002183 31273079

[55] Barbe MT, Reker P, Hamacher S, Franklin J, Kraus D, Dembek TA, Becker J, Steffen JK, Allert N, Wirths J, Dafsari HS, Voges J, Fink GR, Visser-Vandewalle V, Timmermann L. DBS of the PSA and the VIM in essential tremor. Neurology. 2018;91(6):e543–50. 10.1212/WNL.0000000000005956. 29970404 10.1212/WNL.0000000000005956

[56] Shih LC, Pascual-Leone A. Non-invasive brain stimulation for essential tremor. Tremor Other Hyperkinetic Movements. 2017;7:458. 10.7916/D8G44W01. 28373927 10.7916/D8G44W01 PMC5374545

